# Antithrombotic Therapy for Secondary Prevention in Patients with Non-Cardioembolic Stroke or Transient Ischemic Attack: A Systematic Review

**DOI:** 10.3390/life11050447

**Published:** 2021-05-15

**Authors:** Dániel Tornyos, Alexandra Bálint, Péter Kupó, Oumaima El Alaoui El Abdallaoui, András Komócsi

**Affiliations:** Department of Interventional Cardiology, Heart Institute, Medical School, University of Pécs, Ifjúság útja 13, 7624 Pécs, Hungary; tornyos.daniel@pte.hu (D.T.); balint.alexandra@pte.hu (A.B.); kupo.peter@pte.hu (P.K.); oumaimaelalaouielabdallaoui@gmail.com (O.E.A.E.A.)

**Keywords:** stroke, transient ischemic attack, antiplatelet therapy, aspirin, clopidogrel, ticagrelor

## Abstract

Stroke embodies one of the leading causes of death and disability worldwide. We aimed to provide a comprehensive insight into the effectiveness and safety of antiplatelet agents and anticoagulants in the secondary prevention of ischemic stroke or transient ischemic attack. A systematic search for randomized controlled trials, comparing antiplatelet or anticoagulant therapy versus aspirin or placebo among patients with ischemic stroke or transient ischemic attack, was performed in order to summarize data regarding the different regimens. Keyword-based searches in the MEDLINE, EMBASE, and Cochrane Library databases were conducted until the 1st of January 2021. Our search explored 46 randomized controlled trials involving ten antiplatelet agents, six combinations with aspirin, and four anticoagulant therapies. The review of the literature reflects that antiplatelet therapy improves outcome in patients with ischemic stroke or transient ischemic attack. Monotherapy proved to be an effective and safe choice, especially in patients with a high risk of bleeding. Intensified antiplatelet regimens further improve stroke recurrence; however, bleeding rate increases while mortality remains unaffected. Supplementing the clinical judgment of stroke treatment, assessment of bleeding risk is warranted to identify patients with the highest benefit of treatment intensification.

## 1. Introduction

Stroke is the second leading cause of death and one of the leading causes of disability worldwide, accounting for approximately 10% of all mortality events [1]. In our aging society with the increasing incidence of cardiovascular disease (CVD), the rate of cerebrovascular syndromes is also growing [2]. In developed countries, more than 80% of all strokes are of ischemic origin [3]. The risk of recurrence is the highest among cases where a recent stroke or transient ischemic attack (TIA) was left untreated. In about 30% of these cases during the following hours and days, a recurrent stroke leads to the worsening of neurological symptoms or even death [4,5]. Nevertheless, residual disability often puts an enormous strain on our economy [6].

### 1.1. Mechanisms Leading to Stroke

As with CVD, chronic atherosclerosis represents one of the major mechanisms leading to ischemic stroke (IS), via processes of local vascular occlusion and/or thromboembolism. If the atherosclerotic plaque builds up gradually from fatty deposits and cell debris, it can narrow the vessels. Acceleration of ischemia is frequently associated with plaque ruptures, provoking blood clotting. These events may trigger an event sequence, creating a thrombus that can cause local occlusion or embolize the distal segments [4]. Besides atherosclerosis, cardioembolism is the second leading cause of IS. Cardiac emboli are most likely to form in people with certain heart diseases such as atrial fibrillation (AF), heart failure, stenosis, or infections within the valves of the heart. AF as the most frequent cardiac arrhythmia accounts for more than 10% of all IS cases [4]. However, other reasons should be considered especially in younger patients, including carotid-artery dissection, infective endocarditis, and giant cell arteritis [7].

Among AF patients, ischemic risk can be assessed with the help of the CHA_2_DS_2_–VASc score, which consists of the main risk factors of stroke. These factors include congestive heart failure, hypertension, elderly age, diabetes mellitus, prior stroke or TIA or thromboembolism, other vascular diseases, and sex. Guidelines recommend using the CHA_2_DS_2_–VASc score to estimate stroke risk in AF patients, in order to establish the indication of anticoagulation [8]. Despite the overall accepted benefits of the scoring system, some limitations are also associated with its usage. It does not include smoking, which alone doubles the estimated risk of stroke; it also lacks another key factor—high cholesterol levels. These latter risk factors also illustrate that it is possible to dramatically reduce the chance of IS through preventive measures including healthier lifestyle choices or medications [4].

### 1.2. Medical Treatment in Stroke Prevention

Although prevention is necessary for reducing the burden of stroke, the importance of these measures in the survival of cerebral ischemic events remains crucial. Antihypertensive and lipid-lowering therapy, glucose control in patients with diabetes, and smoking cessation are the fundamentals of the prevention. In addition, based on the etiology of the IS, antiplatelet or anticoagulant therapy is inevitable since the coagulation system plays an essential role in stroke pathogenesis [4].

In patients with non-cardioembolic IS or TIA, the clinical guidelines recommend the use of antiplatelet therapy [1,9]. Clinical evidence is the most robust in supporting aspirin (ASA). However, despite its proven benefits, the risk of recurrent stroke remains high in ASA-treated patients [5,10]. Intensification of antiplatelet therapy with more effective agents or with combinations to block multiple platelet activation pathways was tested in numerous randomized controlled trials (RCTs) [5,11,12,13,14]. These strategies appear to be more effective against thrombotic events. Nevertheless, this can come at the cost of an increased risk of hemorrhagic events, including fatal bleeding [5,15].

Importantly, although a certain risk for bleeding may be acceptable, the injured brain parenchyma and fragile cerebral vasculature render patients after IS particularly prone to intracranial bleeding. Thus, it is essential to find an optimal balance between ischemic and bleeding risk.

**Figure 1:**
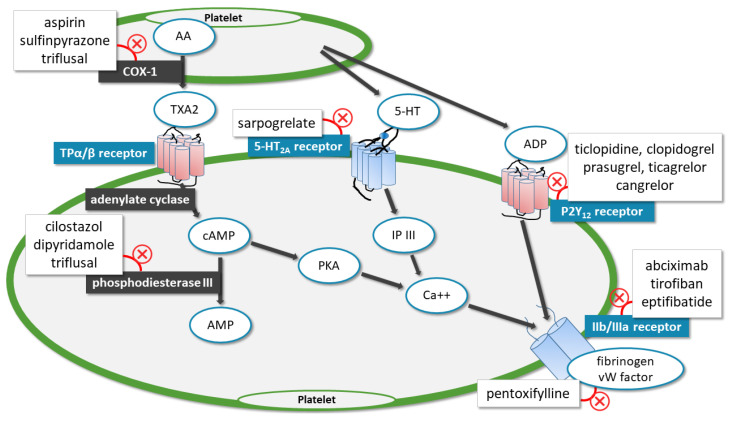
Mechanism of the pharmacological action of the different antiplatelet medications.

In our current era, multiple comparisons of antiplatelet therapies with different mode of actions have been tested in clinical trials, but their results are often conflicting (Figure 1). The main aim of our review was to gain a comprehensive insight into the effectiveness and safety of antiplatelet agents and anticoagulants in the secondary prevention of ischemic stroke based on the results of available RCTs.

## 2. Materials and Methods

**Figure 2:**
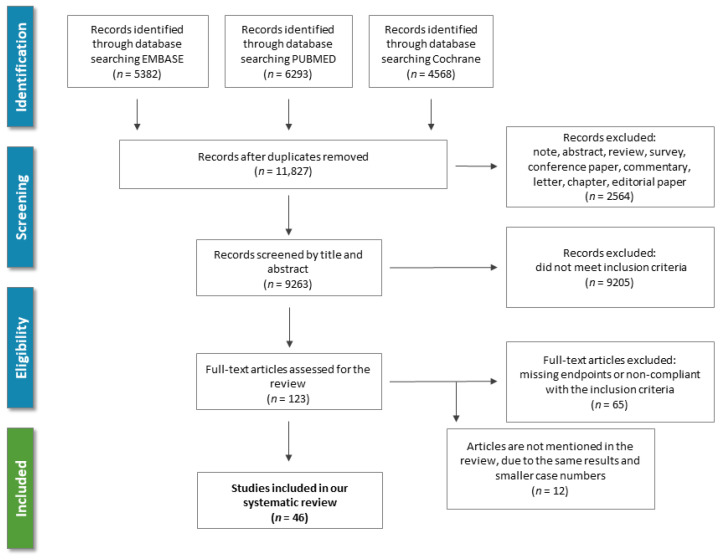
Overview of the study screening and selection process according to PRISMA guidelines.

In order to identify the available literature about the use of antiplatelet or anticoagulant therapy for secondary prevention of IS, a keyword-based search of the medical literature was performed in PubMed (MEDLINE), EMBASE, and Cochrane Library from inception until 1st of January 2021 for articles reporting RCTs of antiplatelet agents in patients with IS or TIA. No language restriction was used. The query included the following medical subject heading (MeSH) terms linked with Boolean operators: “Stroke” [MeSH] OR “Ischemic Attack, Transient” [MeSH] AND “Platelet Aggregation Inhibitors” [MeSH] OR “Anticoagulants” [MeSH]. Furthermore, we searched the reference list of relevant guidelines, reviews, editorials, and studies on this topic. The literature screening process is summarized in Figure 2.

The review included studies that met the following criteria: (A) RCTs evaluating the clinical efficacy and/or safety of an antiplatelet protocol including patients with IS or TIA; (B) one or more treatment arms using an approved platelet inhibitor or combination and a control group consisting of treatment with antiplatelet agents, anticoagulants, or placebo; (C) an indication of the frequency of recurrent strokes during follow-up according to the intention-to-treat analysis, which enables the identification of outcomes attributable to the drugs used. Non-randomized studies, registries, and uncontrolled studies or cohort studies and reviews as well as studies including patients at high risk for cardioembolic stroke were excluded. All relevant sources have been consolidated in a reference manager service (myendnoteweb.com, Clarivate Analytics, Philadelphia, Pennsylvania, USA) to remove duplicates by excluding overlaps between titles, abstracts, authors, and publication years. After removing duplicates, the review authors checked the articles for title, abstract, and full text against our pre-defined eligibility criteria. Each phase was performed in duplicate by two independent investigators, none of whom were blinded to the publication data (DT and AB). Any discrepancies were resolved through arbitration by a third party (AK).

The following details were recorded for each study: name of the study, first author, year of publication, study period, applied platelet aggregation doses, number of patients, length of the treatment period, duration of follow-up, inclusion and exclusion criteria, protocol definitions for recurrent strokes and bleeding events, and characteristics of the patient, including mean age, gender and rate of the following risk factors: diabetes, hypercholesterolemia and high blood pressure (Supplementary Tables S1 and S2).

By the search, 11,827 records were identified and 123 potentially eligible articles were retrieved in full text (Figure 2*)*. Overall, between 1969 and 2020, 46 RCTs comparing 10 antiplatelets including 6 combinations and 4 anticoagulants were included in our review.

## 3. Results

### 3.1. Antiplatelet Agents

#### 3.1.1. Aspirin/Acetylsalicylic Acid

The recent international guidelines recommend ASA as the first choice for preventing recurrent stroke following IS, as ASA’s preventive effect has been proven in numerous RCTs against placebo. ASA irreversibly inhibits cyclooxygenase-1 (COX-1) synthase in platelets and megakaryocytes, and thereby blocks the formation of vasoconstrictive thromboxane A2 (TXA2), which is necessary for platelet aggregation (Figure 1). As it blocks COX-1, it confounds the mucosal barrier and creates gastrointestinal side effects. The first time it was studied was in 1977 for secondary prevention after TIA by Fields et al. In their placebo-controlled trial, they used 1300 mg ASA, and their results showed a significant reduction in recurrent stroke with ASA in cases with multiple attacks, and carotid TIA [16]. Barnet et al. found a 31% reduction in the risk of recurrent stroke or death with 1300 mg ASA [17]. In these two trials, only patients with TIA were included; patients with IS were excluded. On the other hand, in terms of these endpoints, in the Danish and in the Swedish cooperative study, no significant difference was found between 1000/1500 mg ASA and placebo; however, the design of these trials may contribute to the results and decreases the possibility of finding evidence supporting ASA therapy [18,19]. As in several trials, the efficacy of the reduced dose of ASA was proven in cardiovascular diseases. Farrel et al. in the UK-TIA trial compared a higher 1200 mg and a lower 300 mg dose of ASA. They found a 15% reduction with ASA against placebo, but no difference in efficacy between the two doses, and the lower dose was less gastrotoxic [20]. In the ASA arm of the International Stroke Trial (IST), among patients treated with 300 mg ASA, significantly fewer recurrent IS or deaths were seen [21]. Moreover, these results were observed with 160 mg [22], and in the Swedish Aspirin Low-dose Trial (SALT) with 75 mg ASA [23]. These trials support the administration of lower dose ASA as it effectively reduces the ischemic endpoint with no significant excess of hemorrhagic side-effects and other gastrointestinal adverse effects.

#### 3.1.2. Ticlopidine

Ticlopidine is a platelet aggregation inhibitor thienopyridine; it irreversibly blocks the P2Y_12_ component of the adenosine diphosphate (ADP) receptor on the surface of platelets (Figure 1). Its efficacy has been evaluated in four RCTs. Gorelick et al. found no significant difference between the two regimens. Moreover, in the Tokai Panaldine Aspirin Long-term Study (TOPALS), ticlopidine + ASA dual therapy added no benefit compared with ticlopidine alone [24,25]. On the other hand, in the Canadian American Ticlopidine Study (CATS) that randomized patients to 500 mg ticlopidine or placebo, compared with placebo a more than 30% relative risk reduction in the incidence of stroke, myocardial infarction (MI), and vascular death was associated with ticlopidine [26]. Furthermore, in the Ticlopidine Aspirin Stroke Study (TASS), 500 mg ticlopidine was compared with 1300 mg ASA; the results showed a 21% risk reduction in the rates of fatal and non-fatal recurrent stroke with ticlopidine [27]. However, because of its serious side-effects of neutropenia and thrombotic thrombocytopenic purpura, and the availability of newer and safer antiplatelet drugs such as clopidogrel and ticagrelor, the use of ticlopidine remains limited.

#### 3.1.3. Clopidogrel

Clopidogrel is a prodrug, belonging to the family of thienopyridines; after two-step activation, its active metabolite irreversibly blocks the P2Y_12_ component of the ADP receptor (Figure 1). Due to its much more favorable safety profile, it may completely replace ticlopidine. Five RCTs tested clopidogrel monotherapy for secondary prevention of IS. The Clopidogrel Versus Aspirin in Patients at Risk of Ischemic Events (CAPRIE) trial randomized more than 6000 patients after having IS to 325 mg ASA and 75 mg clopidogrel. In the rate of recurrent stroke, MI, or vascular death, they did not find any significant difference between the two groups [28]. Two studies compared clopidogrel with ticlopidine; their results showed fewer bleeding and adverse events with clopidogrel and no significant difference in efficacy [29,30]. In the Prevention Regimen for Effectively Avoiding Second Strokes (PRoFESS) trial, among 20,332 patients, clopidogrel was compared to the combination of ASA + dipyridamole; clopidogrel showed no significant difference in efficacy, but significantly less gastrointestinal bleeding was observed [31]. It must be mentioned that clopidogrel has not been compared with placebo as the efficacy of ASA has been proven before.

#### 3.1.4. Cilostazol

Cilostazol is a selective phosphodiesterase-3 inhibitor that prevents the inactivation of the intracellular second messenger—the cyclic adenosine monophosphate (cAMP)—in platelets, inhibiting platelet aggregation [32,33] (Figure 1). It also augments vasodilation via an endogenous nitric oxide pathway and by decreasing intracellular calcium concentration [32]. Cilostazol also has neuroprotective [34,35] and anti-inflammatory effects [36]. Data are also available showing its potential to delay the onset of atherosclerosis [37], protecting the vascular endothelium, and inhibiting the proliferation of arterial smooth muscle cells [38,39]. The effectiveness of cilostazol monotherapy has been investigated in six RCTs. In the Cilostazol Stroke Prevention Study (CSPS), 1052 patients were randomized either to 200 mg cilostazol or placebo. Among cilostazol-treated patients, a significant relative-risk reduction (41.7%) in recurrent cerebral infarction was seen compared with placebo, and no increase in any bleeding abnormalities was observed [40]. The Cilostazol versus Aspirin for Secondary Ischaemic Stroke Prevention (CASISP) pilot study involved 719 patients, comparing 200 mg cilostazol with 100 mg ASA; their result shows a non-significant 38% reduction in recurrence of stroke (ischemic or hemorrhagic stroke, or subarachnoid hemorrhage). It must be noted that significantly fewer hemorrhagic strokes were observed in the cilostazol group [41]. Its non-inferiority was also observed in the Prevention of Cardiovascular Events in Ischemic Stroke Patients with High Risk of Cerebral Hemorrhage (PICASSO) trial [42]. In the Cilostazol Stroke Prevention Study 2 (CSPS2), 2757 patients were randomized to 200 mg cilostazol or 81 mg ASA, and the mean follow up was 29 months. Cilostazol significantly lowered the risk of recurrent stroke by 25.7%; this result may be due to the significantly fewer hemorrhagic strokes [43]. The superiority of cilostazol in the secondary prevention of IS and the risk of bleeding may be due to its complex mechanism of action which makes it a unique choice among the antiplatelet drugs. However, it must be noted that studies testing cilostazol only included Asian populations, while data regarding other ethnic groups are scarce.

#### 3.1.5. Ticagrelor

Ticagrelor is a potent antiplatelet agent that reversibly binds and inhibits the P2Y_12_ ADP receptor on platelets (Figure 1). Due to the direct acting mechanism, it is not dependent on metabolic activation, in contrast to clopidogrel, thus ticagrelor offers a faster onset and less variable platelet inhibition. Earlier studies have effectively established the efficacy and safety of ticagrelor among patients with acute coronary syndromes (ACSs). However, only a few trials sought to investigate its effectiveness in the secondary prevention of IS. The Acute Stroke or Transient Ischaemic Attack Treated with Aspirin or Ticagrelor and Patient Outcomes (SOCRATES) international trial randomized more than 13,000 patients with IS or TIA, and who did not receive thrombolysis within 24 h after symptoms onset, either to 180 mg ticagrelor or 100 mg ASA after a loading dose on the first day. After 90 days of follow up, they found only a slightly significant difference between the two regimens in terms of recurrent stroke, but no significant difference in bleeding events [10]. Although ticagrelor was not superior to ASA for minor strokes, it seems to be a reasonable alternative in stroke patients who have a contraindication to ASA [1].

#### 3.1.6. Combinations

Antagonizing different steps of platelet aggregation with combinations of drugs through different mechanisms of action can significantly increase antithrombotic efficacy. However, although it may reduce the risk of recurrent IS or TIA, it is important to underline that some combinations may also significantly affect the frequency of bleeding events, resulting in a higher incidence of life-threatening intracranial hemorrhage.

##### Dipyridamole and ASA

Dipyridamole is a phosphodiesterase inhibitor and augments prostacyclin-related platelet aggregation inhibition as it increases the cellular cAMP levels [4] (Figure 1). According to our literature screening, dipyridamole was the first drug studied in the secondary prevention of IS by Acheson et al. in 1969, suggesting that dipyridamole monotherapy was insufficient [44]. On the other hand, the combination of ASA and dipyridamole has been proven effective in the European Stroke Preventive Study (ESPS); the rate of stroke or death was 16% among patients treated with 990 mg ASA and 225 mg dipyridamole compared with 25% among the placebo group [45]. The ESPS2 trial randomized patients into four groups: 50 mg ASA, 400 mg dipyridamole, 50 mg ASA + 400 mg dipyridamole, and placebo. The risk of recurrent stroke was reduced by 18% in the ASA group and 37% with the combined therapy group compared with placebo. Compared with ASA monotherapy, the combination with dipyridamole reduced the risk of recurrent stroke by 23% [46]. However, the results of these studies were debated, as in the first ESPS, the combination therapy was only compared with placebo and in the ESPS2, a significantly lower dose of ASA was tested. In contrast, with ESPS and ESPS2, the results of four RCTs found no additional benefit with the combination of ASA and dipyridamole compared with ASA alone. The first larger trial was the Accidents, Ischemiques Cerebraux Lies a l’Atherosclerose (AICLA) study, by Bousser et al. In their study, they randomized patients to one of the three regimens: 1000 mg ASA, 1000 mg ASA + 225 mg dipyridamole or placebo. In recurrent stroke, they found a significant difference between ASA and placebo but observed no additional benefits with the combination [47]. The same results were observed in the American-Canadian Co-Operative Study, in the European/Australasian Stroke Prevention in Reversible Ischaemia Trial (ESPRIT), and in the Japanese Aggrenox Stroke Prevention versus Aspirin Programme (JASAP study), in which only 81 mg ASA was compared against 50 mg ASA + 400 mg dipyridamole [48,49,50]. As with the ASA and dipyridamole combination, the frequency of adverse effects, such as headache and gastrointestinal symptoms increased, but its efficiency is still not well established due to conflicting results of the available trials.

##### Clopidogrel and ASA

The Management of Atherothrombosis with Clopidogrel in High-risk patients (MATCH) trial compared 75 mg clopidogrel with 75 mg clopidogrel + 75 mg ASA among 7599 patients with recent TIA or IS. According to their results, the combination did not have a significant advantage in ischemic endpoints; on the other hand, the risk of major bleeding was significantly increased [51]. Altogether, ten RCTs have tested the effectiveness and safety of the combination with clopidogrel and ASA against ASA monotherapy. The subgroup analysis of the Clopidogrel for High Atherothrombotic Risk and Ischaemic Stabilisation, Management and Avoidance (CHARISMA) trial enrolled more than 4000 patients after IS or TIA within 5 years. The sub-study of the CHARISMA trial, of IS and TIA patients, showed no significant difference in efficacy, but an increased bleeding risk was seen in the dual therapy group compared with monotherapy [52]. The results of the Secondary Prevention of Small Subcortical Strokes (SPS3) trial were similar to the CHARISMA study, with an increased rate of bleeding events; moreover, a significant increase in the risk of all-cause mortality was observed [53]. These trials tested the dual therapy in the chronic phase after an IS or TIA; however, the risk of stroke recurrence is the highest in the first weeks after IS or TIA. The Fast Assessment of Stroke and TIA to prevent Early Recurrence (FASTER), the Clopidogrel in High-risk patients with Acute Non-disabling Cerebrovascular Events (CHANCE), and the Platelet-Oriented Inhibition in New TIA and Minor Ischemic Stroke (POINT) trials were designed to test the effectiveness of the combination therapy in the acute phase, administering the therapy within the first 24 h after the index event. The FASTER trial enrolled 392 patients within 24 h of symptom onset and randomly assigned them to clopidogrel with a 300 mg loading dose, then a 75 mg daily dose and 81 mg ASA, or to 81 mg ASA only. All patients were followed for 90 days. Unfortunately, the trial was stopped early because of slow recruitment, but the results from the available population showed a 30% risk reduction with the combination therapy, with only a small 1% absolute risk increase for intracranial hemorrhage [54]. The CHANCE trial also included patients within the first 24 h and followed them for 1 year. A 75 to 300 mg loading dose of ASA was given to all patients; the patients in the dual therapy group received a loading dose of 300 mg clopidogrel then 75 mg daily for 90 days, and 75 mg ASA for the first 21 days. The participants in the ASA monotherapy group received 75 mg ASA for 90 days. The risk of recurrent stroke significantly reduced with the dual therapy administered for only 21 days, with no excess in major bleeding events, and this benefit persisted for a duration of 1 year of follow-up [11]. Two smaller RCTs found comparable results with these previous trials [55,56]. Recently, the POINT study assigned patients to either a 600 mg loading dose of clopidogrel followed by 75 mg and 50 to 325 mg ASA or ASA monotherapy, and followed patients for 90 days. Their results showed a significant reduction in the risk of ischemic events with the dual therapy in addition to a significant increase in the risk of major hemorrhage [14]. Both the CHANCE and POINT trials showed that most of the recurrent strokes occur within the first week after an IS or TIA, as incidence curves separate within the first 10 days. As most of the benefits of dual therapy in terms of recurrent strokes occur within the first 10 days, and no further important reduction can be seen after the first 21 days, the recent guidelines recommend dual therapy with clopidogrel and ASA should be initiated within 24 h after onset and maintained for 21 days [1].

##### Ticagrelor and ASA

The effectiveness of the combination of ASA and ticagrelor has been proven in trials including patients with coronary diseases. The Platelet Reactivity in Acute Stroke or Transient Ischaemic Attack (PRINCE) study, a randomized controlled phase 2 trial, involved 675 patients with IS or TIA. Within the first 24 h of symptom onset, patients were randomized to 180 mg ticagrelor + 100 mg ASA or to 75 mg clopidogrel + 100 mg ASA group. At 90 days, they observed a 60% risk reduction in high platelet reactivity in the ticagrelor group. Furthermore, they found a trend in favor of fever recurrent stroke occurred in the ticagrelor group. No difference was observed in the rates of bleeding events between the two groups [10]. The recently published Ticagrelor and Aspirin or Aspirin alone in Acute Ischemic Stroke or TIA (THALES) trial randomized 11,016 patients after non-cardioembolic IS or TIA within 24 h. The patients were assigned to receive a 30-day regimen with 180 mg ticagrelor and 75 to 100 mg ASA or ASA monotherapy. In terms of recurrent IS, they found a 21% risk reduction. On the other hand, the disability rates did not differ, and an extreme 201% risk increase of severe bleeding was observed, and even the rate of mortality was 33% higher in the dual-therapy group. Moreover, in the ticagrelor group, 11 fatal bleeding events occurred, whereas, in the ASA group, only 2 occurred. It must be noted that from 5523 patients, major bleeding complications occurred only in 28 patients in the ticagrelor group, and in the aspirin group of 5493 patients, they occurred only in 7 patients. We can conclude that the benefit seen in the ischemic endpoints may outweigh the disadvantage of bleeding events, although this can only be stated among high ischemic risk patients, as the bleeding risk may exceed this benefit among low-risk patients [5].

### 3.2. Alternative Agents with Antiplatelet Properties

#### 3.2.1. Sulfinpyrazone

Sulfinpyrazone is a uricosuric medication used to treat gout and is also a non-selective COX inhibitor; thus, it inhibits degranulation of platelets and reduces the release of TXA2 (Figure 1). Candelise et al. randomized 124 patients and their study has not shown a significant difference in the efficacy of sulfinpyrazone and ASA [57]. In contrast, Barnet et al. failed to demonstrate its effectiveness, as it did not differ significantly from placebo [16]. Sulfinpyrazone was only tested in patients with TIA, and its utility is questionable.

#### 3.2.2. Pentoxifylline

Pentoxifylline reduces blood viscosity by increasing red cell flexibility, building up intracellular ATP, and decreasing the fibrinogen concentration (Figure 1). Herskovits et al. randomized 66 patients after TIA; they compared 1200 mg pentoxifylline with 1050 mg ASA + 150 dipyridamole. Their results showed significantly lower recurrent TIA in the pentoxifylline group [58]. It must be admitted that whether the low number of included patients and some flaws in the trial design contribute to these results is debated and so far, this remains unconfirmed.

#### 3.2.3. Triflusal

The antithrombotic triflusal chemical structure is related to ASA, but its mechanism of action is more complex. Similarly to aspirin, triflusal irreversibly inhibits COX-1, thus inhibiting TXA2 and preventing platelet aggregation. As it minimally inhibits the endothelial COX-1, it preserves the vascular prostacyclin. Furthermore, it blocks phosphodiesterase, thereby increasing cAMP concentration in platelets and the vascular endothelium, enhancing the antiplatelet effects (Figure 1). Despite its multiple mechanisms of action, its efficacy did not show a significant difference compared to ASA in the Triflusal versus Aspirin for Prevention of Infarction: a Randomized Stroke Study (TAPIRSS) trial, but significantly fewer bleeding events were observed among the triflusal-treated patients [59]. We lack a larger RCT that could justify the superiority of triflusal.

#### 3.2.4. Sarpogrelate

Sarpogrelate is a serotonin receptor antagonist, blocking serotonin-induced platelet aggregation and vasoconstriction (Figure 1). It has been approved to treat patients with peripheral arterial disease in Japan, and in the Republic of Korea. The Sarpogrelate-Aspirin Comparative Clinical study for Efficacy and Safety in Secondary prevention of cerebral infarction (S-ACCESS) trial failed to demonstrate non-inferiority, as the incidence of recurrent stroke was higher in the sarpogrelate group than in the ASA group. Along with its lower efficacy, regarding the safety endpoints, significantly fewer bleeding complications were observed [60].

### 3.3. Anticoagulant Therapy

**Figure 3:**
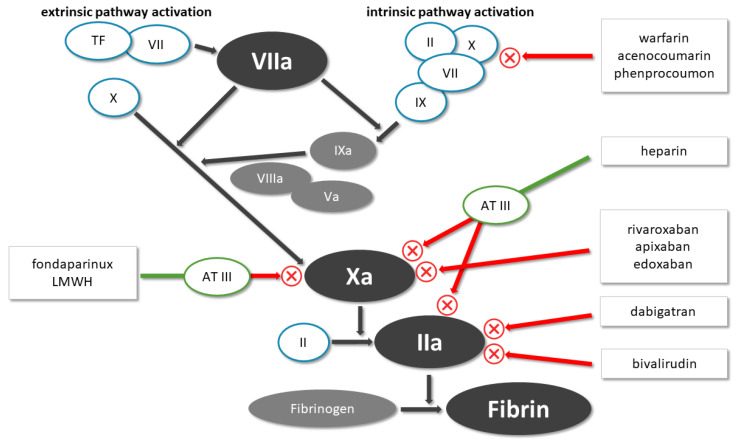
Mechanism of the pharmacological action of the different anticoagulant medications.

Undoubtedly, in the case of cardioembolic stroke, anticoagulant therapy should be chosen for secondary prevention, but in the case of non-cardioembolic stroke, their efficacy has only been studied in a few RCTs so far, with less convincing results (Figure 3).

#### 3.3.1. Warfarin

Warfarin decreases blood clotting by blocking vitamin K epoxide reductase; through this, the active forms of II, VII, IX and X factors cannot be synthetized (Figure 3). Warfarin was tested in a few RCTs in the secondary prevention of IS in people with no history of AF or other cardioembolic causes of stroke. The Warfarin vs. Aspirin Recurrent Stroke Study (WARSS) showed no significant difference between warfarin and ASA in stroke recurrence or in the rate of major hemorrhage [61]. However, the Warfarin-Aspirin Symptomatic Intracranial Disease (WASID) trial was terminated early because of a higher rate of death and major hemorrhage in participants treated with warfarin compared with ASA [62].

#### 3.3.2. Rivaroxaban/Dabigatran

Rivaroxaban and dabigatran are directly acting oral anticoagulants; rivaroxaban inhibits the free factor Xa, and dabigatran is a direct thrombin inhibitor (Figure 3). In the New Approach Rivaroxaban Inhibition of Factor Xa in a Global Trial versus ASA to Prevent Embolism in Embolic Stroke of Undetermined Source (NAVIGATE ESUS) trial, 15 mg rivaroxaban was compared with 100 mg ASA in patients with recent non-cardioembolic IS. The trial was terminated prematurely, as rivaroxaban did not differ in the risk of stroke; nonetheless, the risk of major bleeding increased significantly in the rivaroxaban group [63]. In the Randomized, Double-Blind, Evaluation in Secondary Stroke Prevention Comparing the Efficacy and Safety of the Oral Thrombin Inhibitor Dabigatran Etexilate versus Acetylsalicylic Acid in Patients with Embolic Stroke of Undetermined Source (RE-SPECT ESUS) trial, 300 mg or 220 mg dabigatran was compared with 100 mg ASA. The results showed neither significant benefit in ischemic endpoint, nor a significant difference in major bleeding; however, more clinically relevant non-major bleeding occurred in the dabigatran group [64].

## 4. Discussion

**Figure 4:**
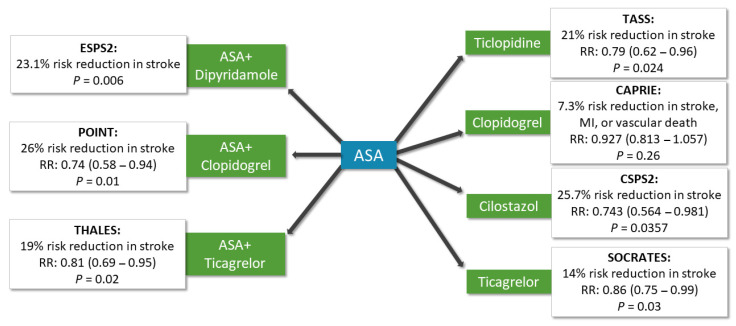
Overview of the most representative study results comparing different platelet aggregation inhibitor regimens to ASA.

Interpretation of the available RCTs is a task with continuously growing difficulty. As the number of trials grows over time, more and more different therapeutic regimens are studied. Therefore, we performed an extensive systematic review, with the aim to comprehensively summarize the available data on antiplatelet therapies in the secondary prevention of stroke. We supplemented the review summarizing pivoting trials that assessed the effectiveness of the different antithrombotic medications compared with ASA (Figure 4, Supplementary Table S3).

In a recent meta-analysis by Xiang et al., 45 RCTs were included. Based on their results, in terms of efficacy and safety, cilostazol and clopidogrel proved to be the more promising choice, as both reduced the risk of recurrent stroke more effectively than ASA [65]. Since this qualitative summary, multiple studies have already been published that may affect the practice of antiplatelet therapy of patients after stroke.

The results of antiplatelet therapy intensification using inhibition of the purinerg P2Y_12_ receptor are, to some extent, conflicting. Two main types of P2Y_12_ receptor inhibitors are used in a clinical setting: prodrug thienopyridines such as ticlopidine, clopidogrel, and prasugrel, and direct-acting antagonists such as ticagrelor and cangrelor. These drugs were tested in several fields of cardiology, and have a well-established role in cases with coronary disease. The combinations of ASA with clopidogrel or ticagrelor belong to standards of treatment in ACSs without or with stent placement [66]. Recurrent stroke was also more effectively alleviated by these intensified antiplatelet schemes; however, in both cases, this effect was counterbalanced with a higher risk of bleeding. Importantly, mortality did not reflect important differences among the applied treatments. In neurology, however, the benefits of more potent inhibitors are inconsistent, as prasugrel significantly increased the rate of intracranial and fatal bleeding in ACS patients with a history of cerebrovascular events [67], while only preliminary data are available with cangrelor in IS cases requiring stenting [68].

The recently published THALES trial perfectly demonstrates the benefits and drawbacks of dual antiplatelet therapy. Ticagrelor when combined with ASA effectively prevented stroke recurrence, yielding a 21% reduction in recurrent stroke compared to ASA, but resulted in a 201% increase in bleeding risk [5] However, a short, intensified therapy may improve the outcomes and results in a balance between bleeding and ischemic endpoints.

Regarding the efficacy and safety aspects, it is important to underline that the early start of antiplatelet therapy has an important impact on the prognosis. As the results of the CHANCE and POINT trials indicate, the benefit of an intensified therapy is most prominent within the first 10 days after the acute event. Afterward, the risk of bleeding events rises and may overcome the ischemic benefit. Bleeding events should not be neglected, as intracranial hemorrhage is associated with a high rate of mortality, especially after an IS stroke, where the injured brain parenchyma is extremely prone to bleeding. Therefore, longer potent dual therapies should be avoided, and only be used after an appropriate risk assessment.

After the acute phase, long-term prevention is needed as the risk of recurrent stroke remains high. Based on the results of many studies, beyond 90 days, monotherapy seems to be the most suitable choice. Besides ASA, cilostazol, clopidogrel and ticagrelor seem to be effective and safe to use for the long term. In addition to ischemic efficacy and bleeding safety, several further aspects need to be considered during therapy selection.

As antiplatelet therapy is continued for years, the cost of the medication plays an important factor. This aspect renders ASA the first choice; however, cost-effectiveness extends beyond the price of a drug, and is measured as life years gained and quality-adjusted life years. Cost-effectiveness analyses of the available trials proved that ASA is more favorable compared with placebo [69]; moreover, analysis of the CHANCE trial found that the 90-day clopidogrel-aspirin increases cost but improves the quality of life, which makes it highly cost-effective compared to ASA [70]. Additionally, the availability of generics makes clopidogrel even financially more attractive. Adverse effects such as gastrointestinal hemorrhage are greater with ASA than clopidogrel. Headache occurs frequently with dipyridamole therapy, which makes it frequently intolerable. Furthermore, serious side effects may occur with ticlopidine such as neutropenia and thrombotic thrombocytopenic purpura. Drug allergy and gastrointestinal intolerance to aspirin may also increase the relevance of other therapeutic choices.

In contrast to the results of the meta-analysis of Xiang et al., the current American Heart Association/American Stroke Association (AHA/ASA) guideline recommends single antiplatelet therapy, most commonly ASA, in patients within 24 to 48 h after onset, and for those treated with thrombolysis, ASA administration delayed until 24 h later. The guideline suggests that, in patients with a contraindication to ASA, administering alternative antiplatelet agents may be reasonable; however, it indicates that the benefit of alternative antiplatelet agents to ASA has not been well established in secondary stroke prevention. After minor non-cardioembolic IS, in patients who did not receive thrombolytic treatment, dual antiplatelet therapy with ASA and clopidogrel is recommended. As the recurrence of stroke is most common in the first month after the index event, the combined antiplatelet treatment should be initiated within 24 h after onset and maintained for 21 days [1,71].

The interpretation of the results of the available RCTs is further complicated by the fact that the severity of stroke varied among the included trials. Most of the studies included patients with minor stroke according to the National Institutes of Health Stroke Scale (NIHSS) score of 3 or less (range 0 to 42, with higher scores indicating more severe stroke), or a high-risk TIA as determined according to a score of 4 or higher on the ABCD^2^ scale (range, 0 to 7, with higher scores indicating a higher risk of stroke) [14]. It is also important to note that multiple mechanisms may lead to the development of stroke, and an important minority of these cases have a cardioembolic origin. Oral anticoagulation remains the treatment of choice of these patients. It is further complicated by the various dosages and treatment durations; therefore, it limits our ability to assess how the differential effects of the dosage of these drugs impact the outcomes. Moreover, the included studies reflected the problems of capturing bleeding events and the lack of one overall accepted bleeding definition system.

## 5. Conclusions

The importance of stroke prevention cannot be underestimated, as stroke is the second leading cause of death and one of the most common causes of disability. In our systematic review, we examined antiplatelet and anticoagulant medical treatments evaluated for secondary prevention of IS or TIA in the available RCTs. Including a wide set of trials, it provides a comprehensive review of the available literature. Antiplatelet therapy improves outcome in patients with IS or TIA. Antiplatelet monotherapy proved to be an effective and safe choice, especially in patients with a high risk of bleeding. Intensified antiplatelet regimens improve stroke recurrence; however, bleeding rate increases, while mortality remains unaffected. Supplementing the clinical judgment of stroke treatment, assessment of bleeding risk is warranted to identify patients with the highest benefit of treatment intensification.

## Data Availability

Data sharing not applicable. No new data were created or analyzed in this study. Data sharing is not applicable to this article.

## Supplementary Materials

The following are available online at https://www.mdpi.com/article/10.3390/life11050447/s1, Table S1. Characteristics of included trials in our systematic review, Table S2. Clinical characteristics of the included patient populations. Table S3. Summary of the most representative studies comparing antithrombotic medications to aspirin.
