# Supplementary material for: Antithrombotic Therapy for Secondary Prevention in Patients with Non-Cardioembolic Stroke or Transient Ischemic Attack: A Systematic Review
Source: Life (Basel). 2021 May 15;11(5):447. doi: 10.3390/life11050447 (PMC8156895; doi:10.3390/life11050447)
Supplement: Supplementary file 1 [file life-11-00447-s001.zip › life-1186868-supplementary.pdf]

life

MDPI

Supplementary material

Review

Antithrombotic Therapy for Secondary Prevention in Patients with Non-Cardioembolic Stroke or Transient Ischemic Attack: A Systematic Review

Dániel Tornyos, Alexandra Bálint, Péter Kupó, Oumaima El Alaoui El Abdallaoui and András Komócsi *

Department of Interventional Cardiology, Heart Institute, Medical School, University of Pécs, Ifjúság útja 13, 7624 Pécs, Hungary; tornyos.daniel@pte.hu (D.T.); balint.alexandra@pte.hu (A.B.); kupo.peter@pte.hu (P.K.); oumaimaelalaouielabdallaoui@gmail.com (O.E.A.E.A.)

* Correspondence: komocsi.andras@pte.hu; Tel.: +36-72-536-001; Fax: +36-72-536-387

Citation: Tornyos, D.; Bálint, A.; Kupó, P.; El Abdallaoui, O.E.A.; Komócsi, A. Antithrombotic Therapy for Secondary Prevention in Patients with Non-Cardioembolic Stroke or Transient Ischemic Attack: A Systematic Review. *Life* **2021**, *11*, 447. https://doi.org/10.3390/life11050447

Academic Editors: Gergely Fehér, Péter Klivényi and Joel Rodriguez-Saldana

Received: 31 March 2021
Accepted: 12 May 2021
Published: 15 May 2021

Publisher's Note: MDPI stays neutral with regard to jurisdictional claims in published maps and institutional affiliations.

Copyright: © 2021 by the authors. Submitted for possible open access publication under the terms and conditions of the Creative Commons Attribution (CC BY) license (https://creativecommons.org/licenses/by/4.0/).

Table S1. Characteristics of included trials in our systematic review:

Trial	Author	Year	Country	Patient cohort	Treatment	No. of Patients	Follow up	Primary outcome	Inclusion criteria	Exclusion criteria
**Aspirin**										
**AITIA**	Fields	1977	USA	TIA	aspirin (1300mg) vs. placebo	88/90	6 months	TIA, stroke, death	• TIA<br>• within 3 months	• bleeding or clotting disorder<br>• history of peptic ulcer<br>• **need for continued use of platelet inhibitors or anticoagulants**
**CCSG**	Barnett	1978	Canada	TIA	aspirin (1300mg) vs. placebo vs. sulfinpyrazone (800mg) vs. aspirin (1300mg) + sulfinpyrazone (800mg	144/139/156/146	mean of 26 months	TIA, stroke, death	• TIA<br>• within 3 months	• coexisting morbid condition explaining their symptoms<br>• likely to die from other illness within 12 months
**Danish Coop**	Sorensen	1983	Denmark	TIA/RIND/AIS	aspirin (1000mg) vs. placebo	101/102	median of 25 months	stroke, death	• TIA<br>• RIND<br>• within 1 month	• residual symptoms from a previous stroke<br>• bad physical condition due to other illness<br>• peptic ulcer<br>• referred to carotid surgery

**Swedish Coop**	Britton	1987	Sweden	AIS	aspirin (1500mg) vs placebo	253/252	2 years	stroke, death	• ischemic stroke<br>• within 1 and 3 weeks	• **atrial fibrillation**<br>• recent myocardial infarction<br>• **other needs for anticoagulant or antiplatelet drugs**<br>• peptic ulcer disease<br>• malignancy<br>• other serious disease
**UK-TIA**	Farrell	1991	UK	TIA/AIS	aspirin (300mg-1200mg) vs placebo	1621/814	mean of 4 years	MACCE	• TIA,<br>• **minor ischemic stroke**<br>• within 3 months<br>• patients were included with cardiac sources of embolism who were not anticoagulated	• history of previous disabling major stroke<br>• due to something other than arterial thromboembolism<br>• renal failure<br>• peptic ulcer<br>• abnormal bleeding<br>• myocardial infarction within 3 months
**IST**	Sandercock	1997	Multiple	AIS	aspirin (300mg) vs. placebo	9719/9714	14 days	death	• ischemic stroke<br>• within 48 hours	• intracranial hemorrhage<br>• gastrointestinal bleeding<br>• **already on long-term anticoagulants**
**CAST**	Chen	1997	China	AIS	aspirin (160mg) vs. placebo	10335/10320	4 weeks	death	• ischemic stroke<br>• within 48 hours	• gastric bleeding<br>• major life-threatening disease<br>• severe pre-existing disability

**SALT**	Elwin	1991	Sweden	TIA/AIS	aspirin (75mg) vs placebo	676/684	median of 32 months	stroke, death	• **minor ischemic stroke**<br>• TIA<br>• within 3 months	• **cardiac source of emboli**<br>• previous or planned carotid surgery<br>• other causes of the symptoms established (arteritis, dissection, migraine, hematological or hyperviscosity disorders, or orthostatically induced symptoms only)<br>• other severe disorders<br>• **need for long-term treatment with anticoagulant or antiplatelet drugs**
**Ticlopidine**										

**AAASPS**	Gorelick	2003	USA	AIS	ticlopidine (500mg) vs. aspirin (650mg)	902/907	average of 1.54 years	MACCE	• ischemic stroke<br>• within 7 days and 90 days	• TIA<br>• subarachnoid hemorrhage<br>• **cardioembolic source**<br>• iatrogenic stroke<br>• postoperative stroke<br>• carotid endarterectomy<br>• hematologic abnormality<br>• bleeding diathesis<br>• active bleeding<br>• severe comorbid condition<br>• dementia other neuro degenerative disease<br>• thrombocytopenia, neutropenia<br>• liver or renal failure
**TOPALS**	Ito	2003	Japan	TIA/AIS	ticlopidine (200mg) vs. ticlopidine (100mg) + aspirin (81mg)	138/132	average of1.59 years	MACCE	• ischemic stroke within 1 and 6 months<br>• TIA within the previous 3 months	• history of cerebral hemorrhage<br>• **cardiogenic cerebral infarction**<br>• severe hepatic or renal dysfunction<br>• bleeding tendency

**CATS**	Gent	1989	North America	AIS	ticlopidine (500mg) vs placebo	525/528	mean of 24 months	MACCE	• ischemic stroke<br>• within 1 week and 4 months	• **cardioembolic cause**<br>• remain bedridden<br>• severe comorbidity<br>• required long-term treatment with anticoagulants or antiplatelet drugs
**TASS**	Hass	1989	North America	TIA/AIS	ticlopidine (500mg) vs. aspirin (1300mg)	1529/1540	1192±460 days	death, stroke	• TIA<br>• **minor ischemic stroke**<br>• within 3 months	• **cardiogenic source**<br>• hematologic disorder<br>• history of peptic ulcer<br>• gastrointestinal bleeding<br>• life threatening disease<br>• **need for continued use of aspirin or anticoagulants**
**Clopidogrel**										

**CAPRIE**	Gent	1996	Multiple	AIS	clopidogrel (75mg) vs. aspirin (325mg)	3233/3198	mean of 1.91 years	MACCE	• ischemic stroke<br>• within 1 week and 6 months	• carotid endarterectomy<br>• intracerebral, intracranial hemorrhage<br>• severe cerebral deficit<br>• severe co-morbidity<br>• scheduled for major surgery<br>• severe renal or hepatic insufficiency<br>• hemostatic disorder or systemic bleeding<br>• thrombocytopenia or neutropenia<br>• history of drug-induced hematologic or hepatic abnormalities<br>• abnormal WBC, differential, or platelet count<br>• **anticipated requirement for long-term anticoagulants**

Fukuuchi 2007	Japan	AIS	clopidogrel (75mg) vs ticlopidine (200mg)	573/578	52 weeks	safety: hematologic changes, hepatic dysfunction, nontraumatic hemorrhage, serious adverse drug reactions<br>sec.: MACCE	• ischemic stroke<br>• from 8 days with no maximum time limit	• **cardioembolic source**<br>• bleeding disorders<br>• risk of bleeding<br>• history of intracranial hemorrhage<br>• current diabetic retinopathy<br>• severe renal or heart disease<br>• uncontrolled hypertension
Uchiyama 2009	Japan	AIS	clopidogrel (75mg) vs ticlopidine (200mg)	939/923	52 weeks	safety: hematologic changes, hepatic dysfunction, and atraumatic serious hemorrhage<br>sec.: MACCE	• ischemic stroke<br>• from 8 days with no maximum time limit	• **cardiac sources of embolism**<br>• TIA after the recent stroke<br>• serious impairment<br>• bleeding disorders<br>• history of intracranial hemorrhage<br>• severe renal<br>• heart disease<br>• uncontrolled hypertension<br>• hepatic dysfunction<br>• thrombocytopenia<br>• leukopenia

**PROFESS**	Sacco	2008	Multiple	AIS	aspirin (50mg) + dipyridamole (400mg) vs. clopidogrel (75mg)	10181/10151	mean of 2.5 years	stroke	• ischemic stroke<br>• within 90 days	• hemorrhagic stroke<br>• brain tumor<br>• stroke induced by a surgical or cardiovascular procedure such as carotid endarterectomy, angiogram or cardiac surgery<br>• active peptic ulcer<br>• history of a hemostatic disorder or systemic bleeding
**Cilostazol**										
**CSPS**	Gotoh	2000	Japan	AIS	cilostazol (200mg) vs. placebo	526/526	mean of 3.2 years	stroke	• ischemic stroke<br>• within 1 and 6 months	• intracranial hemorrhage<br>• **cardioembolic source**<br>• hemostatic disorders<br>• systemic bleeding<br>• malignant tumor<br>• liver cirrhosis<br>• renal failure<br>• heart failure<br>• bedridden
**CASISP**	Huang	2008	China	AIS	aspirin (100mg) vs. cilostazol (200mg)	359/360	1.5 years	stroke	• ischemic stroke<br>• within 1 and 6 months	• history of intracranial or subarachnoid hemorrhage<br>• **cardiogenic embolism**<br>• severe disability<br>• uncontrolled severe comorbidities

**CSPS2**	Shinohara	2010	Japan	AIS	cilostazol (200mg) vs. aspirin (81mg)	1337/1335	mean of 29 months	stroke	• ischemic stroke<br>• within 26 weeks	• **cardioembolic source**<br>• increased risk of hemorrhage<br>• congestive heart failure<br>• peptic ulcer<br>• blood, hepatic, or renal disorders<br>• undergone or scheduled to undergo percutaneous transluminal angioplasty or revascularization
**PICASSO**	Kim	2018	Asia	TIA/AIS	cilostazol (200mg) vs. aspirin (100mg)	755/757	median of 1.9 years	MACCE	• ischemic stroke or TIA with a history of imaging findings of intracerebral hemorrhage or two or more microbleeds<br>• within 180 days	• cerebral hemorrhage within 6 months before study entry<br>• **cardioembolic source**<br>• severe cardiomyopathy<br>• congestive heart failure<br>• myocardial infarction within 4 weeks
**Ticagrelor**										

**SOCRATES**	Johnston	2016	Multiple	TIA/AIS	ticagrelor (180mg) vs. aspirin (100mg)	6589/6610	90 days	MACCE	• **non-severe ischemic stroke**<br>• TIA<br>• within 24 hours	• atrial fibrillation<br>• ventricular aneurysm<br>• **cardioembolic cause**<br>• thrombolysis, thrombectomy<br>• bleeding diathesis<br>• coagulation disorder<br>• history of intracerebral hemorrhage<br>• gastrointestinal bleeding within 6 months<br>• major surgery within 30 days
**Aspirin + Dipyridamole**										
	Acheson	1969	UK	TIA/AIS	dipyridamole (400mg->800mg) vs. placebo	85/84	25 months	stroke, death	• ischemic stroke<br>• TIA<br>• within 5 years	• NA
**ESPS**	Lowenthal	1990	Multiple	TIA/RIND/AIS	aspirin (990mg) + dipyridamole (225mg) vs. placebo	1250/1250	2 years	stroke, death	• TIA<br>• RIND<br>• ischemic stroke<br>• within 3 months	• life-threatening associated disease such as uncontrolled hypertension or diabetes<br>• bleeding diathesis<br>• peptic ulcer

**ESPS 2**	Diener	1996	Multiple	TIA/AIS	aspirin (50mg) vs. dipyridamole (400mg) vs. aspirin (50mg) + dipyridamole (400mg) vs. placebo	1649/1654/1650/1649	2 years	stroke, death	• TIA<br>• ischemic stroke<br>• within 3 months	• gastrointestinal bleeding<br>• bleeding disturbances<br>• **any condition requiring continued use of ASA or anticoagulants**<br>• life-threatening condition
**AICLA**	Bousser	1983	France	TIA/AIS	placebo vs. aspirin (1000mg) vs aspirin (1000mg) + dipyridamole (225mg)	204/198/202	3 years	stroke	• TIA<br>• ischemic stroke<br>• within 1 year	• **atrial fibrillation**<br>• **cardiac valvular disease**<br>• polycythemia<br>• thrombocythemia<br>• estrogen treatment<br>• hemodynamic factors<br>• peptic ulcer<br>• ICA or vertebral artery stenosis
**ACCSG**	Fields	1985	North America	TIA	dipyridamole (300mg) + aspirin (1300mg) vs. aspirin (1300mg)	448/442	median of 25 months	stroke, death	• TIA<br>• within 3 months	• serious concurrent illnesses<br>• take anticoagulants or other drugs that influenced platelet function<br>• carotid endarterectomy<br>• **cardiac source for embolism**<br>• history of peptic ulcer<br>• bleeding or clotting disorder

**ESPRIT**	Halkes	2006	Multiple	TIA/AIS	aspirin (30-325 mg) + dipyridamole (400mg) vs. aspirin (30-325mg)	1363/1376	mean of 3.5 years	MACCE, major bleeding	• TIA<br>• **minor ischemic stroke**<br>• within 6 months	• **cardiac source of embolism**<br>• recent myocardial infarction<br>• carotid stenosis for which carotid endarterectomy or endovascular treatment was planned<br>• blood coagulation disorder<br>• limited life expectancy
**JASAP**	Uchiyama	2011	Japan	AIS	dipyridamole (400mg) + aspirin (50mg) vs. aspirin (81mg)	652/639	mean of 15.6 months	stroke	• ischemic stroke<br>• within 1 week and 6 months	• diagnosis of brain disorders with a bleeding risk<br>• **cardiogenic sources of embolism**<br>• acute coronary syndromes < 6 months<br>• history of peptic ulcer < 3 years<br>• having undergone arterial reconstruction after developing ischemic stroke<br>• severe disability<br>• bleeding or bleeding tendencies<br>• serious cardiac, renal or hepatic disorders<br>• malignant tumor or having received cancer treatment in the past 5 years

**Aspirin + Clopidogrel**									
**MATCH**	Diener	2004	Multiple	TIA/AIS	aspirin (75mg) + clopidogrel (75mg) vs. clopidogrel (75mg)	3797/3802	18 months	MACCE	• ischemic stroke<br>• TIA<br>• within 3 months	• severe comorbid conditions<br>• hepatic insufficiency<br>• peptic ulceration<br>• history of systemic bleeding<br>• bleeding diathesis<br>• coagulopathy<br>• scheduled for major surgery or vascular surgery
**CHARISMA substudy**	Hankey	2011	Multiple	TIA/AIS	clopidogrel (75mg) + aspirin (75-162mg) vs. aspirin (75-162mg)	2157/2163	median of 25 months	stroke	• ischemic stroke<br>• TIA<br>• within 5 years	• taking oral antithrombotic medications or nonsteroidal anti-inflammatory drugs on a long-term basis
**SPS3**	Benavente	2012	Multiple	TIA/AIS	aspirin (325mg) vs. aspirin (325mg) + clopidogrel (75mg)	1503/1517	mean of 3.4 years	stroke	• symptomatic lacunar stroke<br>• within 180 days	• remote cortical infarct<br>• large subcortical infarct<br>• history of intracerebral, intracranial hemorrhage<br>• disabling stroke<br>• surgically amenable ipsilateral carotid artery disease<br>• **cardioembolic cause**

**FASTER**	Kennedy	2007	North America	TIA/AIS	clopidogrel (75mg) + aspirin (81mg) vs. aspirin (81mg)	198/194	90 days	stroke	• TIA<br>• **minor ischemic stroke**<br>• within 24 hours	• intracranial hemorrhage<br>• thrombolysis, thrombectomy<br>• **cardiac source**<br>• acute coronary syndrome<br>• secondary to a procedure<br>• limited life expectancy
**CHANCE**	Wang	2015	China	TIA/AIS	clopidogrel (75mg) + aspirin (75mg) vs. aspirin (75mg)	2584/2586	1 year	stroke	• **minor ischemic stroke**<br>• TIA<br>• within 24 hours	• hemorrhage<br>• vascular malformation<br>• tumor, abscess<br>• other major nonischemic brain disease<br>• **cardiac sources of embolism**<br>• clear indication for anticoagulation<br>• thrombolysis

Fan He	2014	China	TIA/AIS	clopidogrel (75mg) + aspirin (100mg) vs. aspirin (300mg)	321/326	14 days	stroke	• TIA<br>• **minor ischemic stroke**<br>• within 72 hours	• **cardioembolic source**<br>• thrombocytopenia<br>• anticoagulation therapy before stroke onset<br>• definite indication for anticoagulation<br>• current peptic ulceration<br>• history of systemic bleeding<br>• coagulopathy<br>• major surgery or trauma in the previous 3 months<br>• terminal malignancy<br>• serious renal or liver disease<br>• thrombolysis

	Yi	2014	China	AIS	clopidogrel (75mg) + aspirin (200mg) vs. aspirin (200mg)	284/286	30 days	MACCE	• ischemic stroke<br>• within 48 hours	• history of carotid endarterectomy or carotid stent therapy<br>• clinically relevant arrhythmia<br>• atrial fibrillation<br>• liver, renal failure<br>• severe cardiovascular disease<br>• malignancies<br>• thrombocytopenia<br>• anemia<br>• major surgical procedure within 1 week<br>• history of myeloproliferative disorders<br>• heparin-induced thrombocytopenia
**POINT**	Johnston	2018	Multiple	TIA/AIS	clopidogrel (75mg) + aspirin (50-325mg) vs. aspirin (50-325mg)	2432/2449	90 days	MACCE	• TIA<br>• **minor ischemic stroke**<br>• within 12 hours	• thrombolysis<br>• endovascular therapy or endarterectomy<br>• **atrial fibrillation**<br>• **cardiovascular disease in whom anticoagulation would be indicated**
**Aspirin + Ticagrelor**										

**PRINCE**	Wang	2019	China	TIA/AIS	ticagrelor (180mg) + aspirin (100mg) vs. clopidogrel (75mg) + aspirin (100mg)	336/339	90 days	HPR sec.: stroke	• **minor ischemic stroke** • TIA • within 24 hours	• intracranial hemorrhage • acute coronary syndrome • other pathology that could account for the neurological symptoms
**THALES**	Johnston	2020	Multiple	TIA/AIS	ticagrelor (180mg) + aspirin (75-100mg) vs. aspirin (75-100mg)	5523/5493	30 days	stroke, death	• **mild-to-moderate ischemic stroke** • TIA • within 24 hours	• atrial fibrillation • ventricular aneurysm • **cardioembolic cause** • carotid endarterectomy • thrombolysis, thrombectomy • bleeding diathesis • coagulation disorder • history of intracerebral hemorrhage • gastrointestinal bleeding within 6 months • major surgery within 30 days
**Sulfinpyrazone**										

**ATIAIS**	Candelise	1982	Italy	TIA	aspirin (1000mg) vs. sulfinpyrazone (800mg)	63/61	mean of 11.23 months	MACCE	• TIA<br>• within 3 months	• previous peptic ulcer<br>• marked renal or hepatic insufficiency<br>• other life-limiting diseases<br>• **cardiac or hemodynamic causes**<br>• take drugs with antiplatelet or anticoagulant action
**Pentoxifylline**										
	Herskovits	1981	Argentina	TIA	aspirin (1050mg) + dipyridamole (150mg) vs pentoxifylline (1200mg)	36/30	1 year	stroke, TIA, death	• TIA<br>• within 1 month	• gastric symptoms
**Triflusal**										

**TAPIRSS**	Culebras	2004	Multiple	TIA/AIS	triflusal (600mg) vs. aspirin (325mg)	213/216	2 years	MACCE	• TIA<br>• **minor ischemic stroke**<br>• within 15 days and 6 months	• disabling stroke<br>• brain hemorrhage<br>• stroke of non-atherothrombotic cause<br>• **cardioembolic source of stroke**<br>• previous carotid endarterectomy<br>• cognitive impairment<br>• renal or liver failure<br>• moderate or severe heart failure<br>• HIV infection<br>• alcohol or drug abuse<br>• active peptic ulcer<br>• need for long-term anticoagulant, or antiplatelet agents<br>• malignancy with high bleeding risk
**Sarpogrelate**										

**S-ACCESS**	Shinohara	2008	Japan	AIS	sarpogrelate (100mg) vs. aspirin (81mg)	747/752	mean of 1.59 years	stroke	• ischemic stroke<br>• within 6 months	• **cardioembolic stroke**<br>• mRS score of 4 or more<br>• previous or scheduled vascular surgery for cerebral infarction<br>• history of intracranial hemorrhage<br>• systemic bleeding<br>• peptic ulcer<br>• history of bleeding diathesis or coagulopathy<br>• severe complications such as cardiac, renal, hepatic, and blood disorders<br>• treatment for malignancy within the past 5 years
**Warfarin**										
**WARSS**	Mohr	2001	USA	AIS	warfarin vs. aspirin (325mg)	1103/1103	2 years±1 month	ischemic stroke, death	• ischemic stroke<br>• within 30 days	• due to a procedure<br>• carotid stenosis for which surgery was planned<br>• **cardioembolic source**

**WASID**	Chimowitz	2005	North America	TIA/AIS	aspirin (325-1300mg) vs warfarin	280/289	mean of 1.8 years	stroke, brain hemorrhage, CV death	• TIA<br>• **nondisabling stroke**<br>• within 90 days	• 50 to 99 percent stenosis of the extracranial carotid artery<br>• non-atherosclerotic stenosis of an intracranial artery<br>• **cardiac source of embolism (e.g., atrial fibrillation)**
**Rivaroxaban**										
**NAVIGATE ESUS**	Hart	2018	Multiple	AIS	rivaroxaban (15mg) vs aspirin (100mg)	3609/3604	median of 11 months	stroke, bleeding	• **minor ischemic stroke**<br>• between 7 days and 6 months	• extracranial vessel atherosclerosis causing more than 50% luminal stenosis<br>• **cardiac source of embolism**<br>• severely disabling stroke<br>• indication for anticoagulation or for antiplatelet therapy<br>• major bleeding within the previous 6 months<br>• previous nontraumatic intracranial hemorrhage
**Dabigatran**										

**RE-SPECT ESUS**	Diener	2019	Multiple	AIS	dabigatran (220-300mg) vs. aspirin (100mg)	2695/2695	median of 19 months	stroke, bleeding	• **minor ischemic stroke**<br>• within 3 months	• **cardioembolic source**<br>• intracerebral hemorrhage<br>• increased risk of bleeding<br>• mRS score of ≥4 at the time of randomization<br>• other specific stroke etiology (e.g., cerebral arteritis or arterial dissection, migraine/vasospasm, drug abuse)<br>• renal failure

Abbreviations: AIS: acute ischemic event, ASA: aspirin, CV: cardiovascular, HPR: high platelet reactivity, ICA: internal carotid artery, MACCE: major adverse cardiac and cerebrovascular events, MES: microembolic signals, MRI: magnetic resonance imaging, mRS: modified Rankin Scale, NA: not applicable, RIND: reversible ischemic neurological deficit, TIA: transient ischemic attack, WBC: white blood count.
cardiogenic source: mitral valve stenosis, prosthetic valve, endocarditis, valvular vegetations, myocardial infarction within 6 weeks, ventricular aneurysm intraventricular or intraatrial blood clots, mitral valve prolapse, atrial fibrillation, sick sinus syndrome, idiopathic cardiomyopathy, atrial myxoma or other cardiac tumors.

Table S2. Clinical characteristics of the included patient populations:

Acronym year author	Blinding	Patient cohort	Lenght of treatment period	No. sample size	No. (%) female sex	Mean age (years)	No. (%) prior TIA	No. (%) prior stroke	No. (%) hypertension	No. (%) PAD	No. (%) prior MI	No. (%) DM	No. (%) dyslipidaemia
**Aspirin**													
**AITIA 1977 Fields**	double blind	TIA	6 months	178	60 (34)	NA	NA	16 (9)	84 (47)	16 (9)	33 (18)	25 (14)	NA
**CCSG 1978 Barnett**	double blind	TIA	mean of 26 months	585	179 (31)	NA	NA	NA	NA	NA	NA	NA	NA
**Danish Coop 1983 Sorensen**	double blind	TIA/RIND/AIS	median of 25 months	203	55 (27)	59	NA	11 (5)	55 (27)	30 (15)	18 (8)	NA	59 (29)
**Swedish Coop 1987 Britton**	double blind	AIS	2 years	505	192 (38)	68	40 (8)	55 (11)	232 (46)	45 (9)	50 (10)	86 (17)	10 (2)
**UK-TIA 1991 Farrell**	double blind	TIA/AIS	mean of 4 years	2435	656 (27)	60	NA	81 (3)	955 (39)	298 (12)	242 (10)	106 (4)	856 (35)
**IST 1997 Sandercock**	open label	AIS	14 days	19433	NA	NA	NA	NA	NA	NA	NA	NA	NA
**CAST 1997 Chen**	double blind	AIS	4 weeks	21106	7739 (37)	63	NA	NA	NA	NA	NA	NA	NA
**SALT 1991 Elwin**	double blind	TIA/AIS	mean of 30 and 27 months	1360	470 (34)	67	128 (9)	124 (9)	632 (46)	111 (8)	149 (11)	175 (13)	NA
**Ticlopidine**													

**AAASPS 2003 Gorelick**	double blind	AIS	average of 1.54 years	1809	967 (53)	61	NA	NA	1539 (85)	NA	170 (9)	738 (41)	697 (38)
**TOPALS 2003 Ito**	open label	TIA/AIS	average of 1.59 years	270	95 (35)	67	NA	NA	128 (47)	NA	NA	61 (23)	27 (10)
**CATS 1989 Gent**	double blind	AIS	mean of 18 months	1053	406 (38)	65	183 (17)	821 (78)	710 (67)	110 (10)	179 (17)	331 (31)	NA
**TASS 1989 Hass**	double blind	TIA/AIS	818±592 days	3069	1082 (35)	63	NA	295 (10)	1204 (39)	447 (14)	514 (17)	597 (19)	1122 (36)
**Clopidogrel**													
**CAPRIE 1996 Gent**	double blind	AIS	mean of 1.63 year	6431	2346 (36)	65	1221 (19)	1157 (18)	4179 (65)	NA	772 (12)	1639 (25)	2411 (0.37)
**2007 Fukuuchi**	double blind	AIS	52 weeks	1151	311 (27)	64	NA	NA	780 (68)	NA	NA	434 (38)	222 (19)
**2009 Uchiyama**	double blind	AIS	26 -52 weeks	1862	529 (28)	64	NA	NA	1304 (70)	NA	NA	410 (22)	661 (35)
**PROFESS 2008 Sacco**	double blind	AIS	1.5-4.4 years	20332	7319 (36)	66	1769 (8)	3709 (18)	15045 (74)	609 (3)	1362 (7)	5743 (28)	9474 (46)
**Cilostazol**													
**CSPS 2000 Gotoh**	double blind	AIS	663±461 days	1052	684 (65)	65	NA	NA	636 (60)	NA	NA	258 (25)	252 (24)
**CASISP 2008 Huang**	double blind	AIS	12-18 months	719	225 (31)	60	NA	NA	569 (79)	NA	NA	131 (18)	211 (29)
**PICASSO 2018 Kim**	double blind	AIS	median of 1.9 years	1512	575 (38)	65	NA	NA	1347 (89)	NA	NA	485 (32)	661 (44)
**CSPS2 2010 Shinohara**	double blind	AIS	1-5 years	2672	756 (28)	63	NA	NA	1967 (74)	NA	NA	775 (29)	1159 (43)

**Ticagrelor**													
**SOCRATES 2016 Johnston**	double blind	TIA/AIS	3 months	13199	5483 (42)	66	856 (6)	1593 (12)	9730 (74)	NA	548 (4)	3212 (24)	5028 (38)
**Aspirin + Dipyridamole**													
**1969 Acheson**	double blind	TIA/AIS	25 months	169	52 (31)	58	NA	NA	97 (57)	NA	NA	NA	NA
**ESPS 1990 Lowenthal**	double blind	TIA/RIND/AIS	2 years	2500	1050 (42)	63	NA	NA	916 (36)	NA	NA	NA	NA
**ESPS 2 1996 Diener**	double blind	TIA/AIS	2 years	6602	2774 (42)	67	NA	NA	3997 (60)	1454 (22)	NA	1011 (15)	1509 (23)
**AICLA 1983 Bousser**	double blind	TIA/AIS	3 years	604	181 (30)	63	NA	NA	380 (63)	42 (7)	42 (7)	132 (22)	157 (26)
**ACCSG 1985 Fields**	double blind	TIA	median of 25 months	890	294 (33)	63	NA	116 (13)	423 (48)	69 (8)	134 (15)	132 (15)	NA
**ESPRIT 2006 Halkes**	open label treatment / blinded endpoints	TIA/AIS	mean of 3.5 years	2739	950 (35)	63	NA	314 (11)	1631 (59)	128 (5)	184 (7)	512 (19)	1272 (46)
**JASAP 2011 Uchiyama**	double blind	AIS	447-471 days	1294	369 (28)	66	NA	NA	1143 (88)	NA	NA	523 (40)	856 (66)
**Aspirin + Clopidogrel**													
**MATCH 2004 Diener**	double blind	TIA/AIS	18 months	7599	2821 (37)	66	1442 (19)	1981 (26)	5945 (78)	776 (10)	363 (5)	5197 (68)	4280 (56)

**CHARISMA substudy 2011 Hankey**	double blind	TIA/AIS	median of 25 months	4320	1576 (36)	64	1410 (32)	3332 (77)	3295 (76)	259 (6)	NA	1253 (29)	NA
**SPS3 2012 Benavente**	double blind	TIA/AIS	mean of 3.4 years	3020	1117 (37)	63	452 (15)		2264 (75)	NA	NA	1102 (36)	NA
**FASTER 2007 Kennedy**	double blind	TIA/AIS	90 days	392	185 (47)	68	63 (16)	29 (7)	198 (50)	8 (2)	19 (4)	42 (10)	28 (7)
**CHANCE 2015 Wang**	double blind	TIA/AIS	90 days	5170	1750 (34)	62	174 (3)	1033 (20)	3399 (66)	NA	96 (2)	1093 (21)	573 (11)
**2014 Fan He**	open label	TIA/AIS	14 days	647	279 (43)	62	221 (34)		437 (67)	NA	NA	266 (41)	NA
**2014 Yi**	open label	AIS	30 days	570	257 (45)	70	NA	NA	414 (73)	NA	7 (1)	215 (38)	NA
**POINT 2018 Johnston**	double blind	TIA/AIS	90 days	4881	2195 (45)	65	NA	NA	3373 (69)	NA	NA	1340 (27)	NA
**Aspirin + Ticagrelor**													
**PRINCE 2019 Wang**	open label treatment / blinded endpoints	TIA/AIS	3 months	675	181 (27)	61	18 (3)	121 (18)	411 (61)	NA	NA	164 (24)	41 (6)
**THALES 2020 Johnston**	double blind	TIA/AIS	30 days	11016	4279 (39)	65	515 (5)	1815 (17)	8520 (77)	NA	NA	3142 (29)	NA
**Sulfinpyrazone**													
**ATIAIS 1982 Candelise**	double blind	TIA	mean of 11.23 months	124	38 (31)	54	NA	NA	58 (47)	11 (9)	NA	9 (7)	56 (45)

**Pentoxifylline**													
**1981 Herskovits**	open label	TIA	1 year	66	16 (24)	60	NA	NA	39 (59)	NA	NA	11 (16)	48 (72)
**Triflusal**													
**TAPIRSS 2004 Culebras**	double blind	TIA/AIS	mean of 586 days	429	136 (32)	65	67 (16)	55 (13)	303 (71)	15 (3)	24 (5)	78 (18)	160 (37)
**Sarpogrelate**													
**S-ACCESS 2008 Shinohara**	double blind	AIS	mean of 580 days	1499	423 (28)	65	NA	200 (13)	1037 (69)	NA	NA	419 (28)	593 (39)
**Warfarin**													
**WARSS 2001 Mohr**	double blind	AIS	2 years±1 months	2206	897 (41)	63	629 (29)		1499 (68)	NA	NA	705 (32)	NA
**WASID 2005 Chimowitz**	double blind	TIA/AIS	mean of 1.8 years	569	219 (38)	64	NA	138 (24)	477 (84)	NA	NA	216 (38)	391 (69)
**Rivaroxaban**													
**NAVIGATE ESUS 2018 Hart**	double blind	AIS	median of 11 months	7213	2777 (38)	67	1263 (17)		5585 (77)	NA	NA	1806 (25)	NA
**Dabigatran**													
**RE-SPECT ESUS 2019 Diener**	double blind	AIS	median of 19 months	5390	1987 (37)	64	975 (18)		3981 (74)	NA	340 (6)	1224 (23)	3043 (56)

Abbreviations: AIS: acute ischemic event, DM: diabetes mellitus, MI: myocardial infarction, NA: not applicable, PAD: peripheral artery disease, RIND: reversible ischemic neurological deficit, TIA: transient ischemic attack.

Table S3. Summary of the most representative studies comparing antithrombotic medications to aspirin:

RCT	ASA vs.	Event	RR	P	Administration time	Follow up
**CAST**	Placebo	IS	NA	0.01	within 48 hours	4 weeks
**TASS**	Ticlopidine	S	0.79 (0.62 – 0.96)	0.024	within 3 months	mean of 3.26 years
**CAPRIE**	Clopidogrel	S+MI+VD	0.927 (0.813 – 1.057)	0.26	within 1 week and 6 months	mean of 1.91 years
**CSPS2**	Cilostazol	S	0.743 (0.564 – 0.981)	0.0357	within 26 weeks	mean of 29 months
**SOCRATES**	Ticagrelor	S	0.86 (0.75 – 0.99)	0.03	within 24 hours	90 days
**ESPS2**	ASA + Dipyridamole	S	NA	0.006	within 3 months	2 years
**POINT**	ASA + Clopidogrel	S	0.74 (0.58 – 0.94)	0.01	within 12 hours	90 days
**THALES**	ASA + Ticagrelor	S	0.81 (0.69 – 0.95)	0.02	within 24 hours	30 days
**WASID**	Warfarin	S	1.20 (0.82 – 1.75)	0.34	within 90 days	mean of 1.8 years
**NAVIGATE ESUS**	Rivaroxaban	S	1.08 (0.87 – 1.34)	NA	within 7 days and 6 months	median of 11 months
**RE-SPECT ESUS**	Dabigatran	S	0.85 (0.69 – 1.03)	0.10	within 3 months	median of 19 months

Abbreviations: ASA: aspirin, acetylsalicylic acid, IS: ischemic stroke, MI: myocardial infarction, RCT: randomized controlled trial, S: ischemic + hemorrhagic stroke, VD: vascular death.
